# Lack of Adoption of a Mobile App to Support Patient Self-Management of Diabetes and Hypertension in a Federally Qualified Health Center: Interview Analysis of Staff and Patients in a Failed Randomized Trial

**DOI:** 10.2196/humanfactors.7709

**Published:** 2017-10-03

**Authors:** Kathleen Thies, Daren Anderson, Benjamin Cramer

**Affiliations:** ^1^ Community Health Center, Inc. Weitzman Institute Middletown, CT United States; ^2^ Western Michigan University Kalamazoo,, MI United States

**Keywords:** telehealth, mobile health, mHealth, underserved patients, HIT, usability

## Abstract

**Background:**

Thousands of mobile health (mHealth) apps have been developed to support patients’ management of their health, but the effectiveness of many of the apps remains unclear. While mHealth apps appear to hold promise for improving the self-management of chronic conditions across populations, failure to balance the system demands of the app with the needs, interests, or resources of the end users can undermine consumers’ adoption of these technologies.

**Objective:**

The original aim of this study was to evaluate the effectiveness of a commercial mHealth app in improving clinical outcomes for adult patients in a Federally Qualified Health Center (FQHC) with uncontrolled diabetes and/or hypertension. Patients entered clinical data into the app, which also supported messaging between patients and providers. After a 4-month period of vigorous recruitment, the trial was suspended due to low enrollment and inconsistent use of the app by enrolled patients. The project aim was changed to understanding why the trial was unsuccessful.

**Methods:**

We used the user-task-context (eUTC) usability framework to develop a set of interview questions for patients and staff who were involved in the trial. All interviews were done by phone and lasted 20 to 30 minutes. Interviews were not recorded.

**Results:**

There was a poor fit between the app, end users, and recruitment and treatment approaches in our setting. Usability testing might have revealed this prior to launch but was not an option. There was not sufficient time during routine care for clinical staff to familiarize patients with the app or to check clinical data and messages, which are unreimbursed activities. Some patients did not use the app appropriately. The lack of integration with the electronic health record (EHR) was cited as a problem for both patients and staff who also said the app was just one more thing to attend to.

**Conclusions:**

This brief trial underscores the pitfalls in the utilization of mHealth apps. Effective use of mHealth tools requires a good fit between the app, the users’ electronic health (eHealth) literacy, the treatment approach, staff time, and reimbursement for services. The last 3 are contextual factors of the setting that affected the adoption of the app and context is an important factor in implementation science. We recommend that researchers address contextual factors in the trial and adoption of mHealth technologies.

## Introduction

Thousands of mobile health (mHealth) apps have been developed to support patient’s management of their health [1,2], but their effectiveness remains unclear [3]. Despite reporting improved outcomes, many studies of mHealth apps are not adequately powered, have poor rates of retention, or have a high risk of bias in their methods [4]. While mHealth technologies appear to hold promise for improving the self-management of chronic conditions across populations [5-8], failure to balance the system demands of the app with the needs, interests, or resources of the end users can undermine consumers’ adoption of these technologies [9-12].

This was the case in a pilot trial of a mHealth app with patients in a Federally Qualified Health Center (FQHC) who had uncontrolled diabetes and/or hypertension. The original aims of this trial were to evaluate the effectiveness of the app in improving clinical outcomes and the app’s usability. However, after a 4-month period of vigorous recruitment, the trial was suspended due to low enrollment and the project aim was changed to understanding why the trial was unsuccessful. This paper briefly describes the original study to provide context and then describes the secondary study in which we focused on usability [11,12]. Lessons learned and recommendations are also discussed.

## Methods

### Original Study Design

#### Setting

Community Health Center, Inc. (CHCI) is a multisite FQHC and level III patient-centered medical home (PCMH) in Connecticut providing comprehensive primary medical, dental, and behavioral health care to 140,000 medically underserved patients in over 200 delivery locations; 75% of patients have fee-for-service Medicaid insurance. CHCI has a fully integrated electronic health record (EHR) and uses a team-based, integrated model of care. Each primary care provider (PCP) is supported by 1 medical assistant (MA) and a primary care registered nurse (RN). One nurse generally supports 2 PCPs. Most routine management of hypertension and type 2 diabetes is carried out by PCPs supported by the nurses and complemented by on-site diabetes and nutrition education. This study was approved by the Institutional Review Board (IRB) of CHCI.

#### Intervention

The app used for the intervention was developed by a commercial vendor to run on Mac and Windows platforms. CHCI was not involved in the development and testing of the app. Adoption of the app by CHCI was prompted by 2 studies that reported improved clinical outcomes in patients who used the app to manage their diabetes and/or hypertension [13,14].

The app provides a platform for active collaboration between patients and their primary care team between office visits. After working with the care team to develop personal goals and strategies for managing their chronic conditions, patients then enter clinical information into the app, such as weight, blood pressure, and blood glucose levels, and frequency of adhering to a treatment regimen, such as medication, diet, or exercise. The delivery interface includes charts that display trends in clinical data over time and graphics indicating patient progress toward goals. Members of the patient’s care team track patients’ progress toward their goals and exchange messages providing encouragement and suggestions and answering non-pressing questions.

The app can be accessed by downloading it on a mobile phone or accessing it on the vendor’s website. The interface display is the same between the platforms. The end-users, which are the patients and care team members, require a profile that is visible to the vendor. The vendor created the profiles for the care team (ie, the primary care provider, nurse, and research assistants). Staff created the profiles for the patients. All data is stored in the vendor’s warehouse.

#### Population

Patients eligible for the trial were aged 18 and over and had at least 1 visit to CHCI in the previous 6 months. Uniform Data System (UDS) measures, which CHCI reports, were chosen as the criteria for uncontrolled hypertension and diabetes because these criteria are used to develop clinical dashboards that identify patients with uncontrolled chronic disease [15]. Thus, uncontrolled hypertension was defined as having a blood pressure of either 140/90 or higher on record in the EHR at the time of the patient’s last visit [15]. Similarly, uncontrolled diabetes was defined as a recorded glycated hemoglobin (HbA1c) greater than 9% at the patient’s last visit [15].

#### Recruitment

Eligible patients were identified through a chronic conditions dashboard based on the UDS measures, and were recruited on a rolling basis from 1 CHCI primary care site. The plan was for the PCP, nurses, and research assistants to approach patients in person in the clinic, explain the app, and assist with downloading the app as needed. Research assistants also called patients, targeting those with upcoming appointments, providing assistance by phone. Patients were informed that the app is not to be used for emergencies or prescription refills and that staff would respond to messages within 24 hours of posting.

#### Original Study Design Results

Over a 4-month period, about 90 patients were approached either by phone or in person. A profile was created for 22 interested patients: 6 (27%, 6/22) patients had diabetes, 4 (18%, 4/22) had hypertension, and the remaining 12 (55%, 12/22) were diagnosed with both. The average age was 50 (range 31 to 69); 12 (54.5%, 12/22) were female and 10 (45%, 10/22) were male. Of the 22 patients, 7 (32%, 7/22) did not download the app. Of the remaining 15 patients who downloaded the app, 7 (47%, 7/15) were female. The patients who declined participation said they were not interested, didn’t have time to learn about the app, and/or didn’t have a mobile phone or computer. The trial was suspended due to low enrollment and inconsistent use of the app by those who did enroll.

### Secondary Study

#### Overview

After the failure to recruit and retain sufficient numbers of patients to complete the trial, we evaluated the usability of the app. In light of the unsuccessful trial, we used a realist evaluation approach, which challenged us to more closely examine the unspoken assumptions behind our initiative [16]. Based on our experience, we were sure that most CHCI patients had a mobile phone; mobile phone ownership is prevalent across underserved populations [7]. But we assumed that patients would perceive that the app would help them their manage hypertension and diabetes and have the technology and proficiency to use it. Similarly, we assumed staff would be able to recruit patients, as well as use the app to collaborate with them.

We used the user-task-context (eUTC) usability framework to evaluate the results of our trial [11,12]. The eUTC has 3 dimensions and 7 domains for understanding the needs of users of eHealth technology, all within the context of healthcare (Textbox 1). In the eUTC framework, the “user” dimension refers to people who use the app, that is, patients and members of the healthcare team. The “task” dimension represents the activities involved in using technology, such as how many steps are required and how difficult it may be to navigate. Finally, the “interface” dimension is where user and task meet, and users sense the ease of and benefits of using the technology.

The eUTC is enhanced by the inclusion of eHealth literacy in the framework [17]. eHealth literacy is defined as “the ability to seek, find, understand, and appraise health information from electronic sources and apply the knowledge gained to addressing or solving a health problem” [18]. However, users of technology vary in their levels of electronic health (eHealth) literacy, such as health literacy, traditional literacy, numeracy, computer literacy, media literacy, science literacy, and information literacy [19].

It is important to note that the eUTC usability framework is used by developers of eHealth technologies in the process of designing and testing applications for consumer use and not after their implementation with end-users [11,17]. However, as CHCI was not involved in the development of the app in our pilot study, we used the eUTC framework as an evaluation tool after the fact to guide interviews with both sets of end-users: patients and staff.

#### Intervention

The three dimension framework noted above guided the questions asked during semi-structured interviews of participants [11,12]. Textbox 2 provides sample questions that were asked of patients; questions for staff mirrored those asked of patients. Of the 15 patients enrolled in the app, 8 (53%) agreed to be interviewed, as well as 1 PCP, 2 nurses, and 2 research assistants. All interviews were done by phone and lasted 20 to 30 minutes. Interviews were not recorded.

**Three dimension framework for understanding needs of users of technology.**

Dimensions

- User dimension
  - Knowledge about one's own health
  - Ability to use information
  - Ability to engage with technology
- Interface
  - Feel that technology is beneficial
  - Feel in control and secure when using technology
- Task dimension
  - Access to technologies that work
  - Access to technologies that suit individual needs

**Sample questions for patients.**

Questions

- User dimension questions
  - Tell us how you deal with your [chronic disease].
  - How do you get most of your information about your hypertension?
  - What kinds of technologies do you use? Internet? Mobile phone?
- Interface questions
  - Did you think the app was beneficial? How did your provider’s opinion about the app affect your use of it?
  - How do you feel when you use technology? When you used the app?
  - What would be an ideal app for these purposes?
- Task dimension questions
  - How did you use the app?
  - What do you think about how it worked?
  - Did the app meet your needs?

## Results

### Quantitative Data

As noted earlier, a profile was created for 22 interested patients, 7 (32%, 7/22) of whom did not download the app. The 15 patients who enrolled in the app sent a total of 139 messages, an average of 19 per patient (range 0 to 39). Two users accounted for about half (48.2%, 67/139) of the messages. Staff sent 141 messages, spending just under 7 hours in total on the app during the trial, or about 26 minutes per patient. Two patients entered their blood pressure, as taken at home, in the first week of using the app, and both entered blood pressure again a week later, with no change. One patient entered weight twice and 5 patients entered fasting blood glucose (FBG) levels twice. Only 1 saw a drop in FBG levels, from 120 to 79. These 8 patients did not enter any further data, and the remaining 7 patients did not enter any information at all.

### Qualitative Data

#### Patient Interviews

##### User Dimension

All 8 patients who were interviewed had a mobile phone, 6 (75%, 6/8) of which were mobile phones with app capabilities and 4 (67%, 4/6) used apps on their mobile phones. It was found that 63% (5/8) had a computer and 3 (38%, 3/8) reported using it routinely. For self-management of their chronic condition, most patients did “home monitoring, years of little calendars…take medication, eat right, exercise and lose weight.” Others (38%, 3/8) relied on their providers as “they tell me what to do.” The extent to which these patients followed through with these self-management strategies is unclear given that they had been identified as having uncontrolled disease.

##### Task Dimension

Several patients compared the app to the patient portal that is part of the EHR platform used by CHCI. Some found the app easier to use, while others did not.

[The portal] is confusing…[the app] is much easier to use than the portal.

...[the app is] too complicated, didn't want to deal with it.

While some reported that they got a quicker response to messages sent to the provider, others did not.

got answers quicker with [the app].

[I am]…frustrated that [provider] has not been there like he was [on portal]. I could ask a question in the morning and get a response by 4:00 pm.

Although patients were not expected to use both the app and paper logs of their blood pressure or blood glucose readings, most noted that the app “has become another step…one more thing,” whereas paper logs required fewer steps. Several patients used the app to request medication refills, even though they had been advised otherwise. Some patients did not know how to download a free app when they had no credit card on file with the phone’s service provider.

##### Interface Dimension

Several patients stated that the ideal app they would be most comfortable with would be “simple and easy”. However, they did not elaborate on what that would look like, other than to note that the app in question did not quite meet those criteria. Of the patients, 3 (38%, 3/8) expressed concerns about the privacy of their health information on the app and7 (88%, 7/8) wanted an app that connected to their EHR because:

the information would be private and safe….everything would be in one place [such as, requests for medication refills]…all part of your record.

All patients indicated ease of contact with providers is an important feature of any health-related technology.

Reaching the doctor anytime I could…I just need to see my doctor and be able to talk to them when I need to.

Others (38%, 5/8) mentioned other types of functionality.

Liked the indication [in the app] that there is a message

from provider

...if it had a social media component, without HIPAA.

a reference to the Health Information Portability and Accountability Act, which requires that personal health information not be shared with unauthorized persons

would [like it to] remind me to take my medication.

#### Staff Interviews

##### User Dimension

All of the staff were expert users of technology for both professional and personal purposes. The nurses and PCP reported that most disease management occurs in person with the patient during provider and/or nurse visits and by telephone. They routinely used dashboards to identify patient blood pressure or blood glucose/HbA1c levels, enroll high risk patients in care coordination, and help patients develop action plans and home monitoring.

##### Task Dimension

The staff noted that many patients who did not enroll did not have access to technology, were not comfortable with technology, or faced barriers to its use.

Many patients have a flip phone or don’t use the phone other than for calls…others forgot password [to app].

Lack of time was a major factor in usage. Downloading the app in the clinic took “too much time, the [Internet] was slow [in some parts of the building]”, and when patients couldn’t download the app right away they lost interest. There was not enough time for staff to explain the app during a 20-minute visit in which their priority was an acute illness, and not their chronic condition. Patients did not want to wait after their visits for the nurse or research assistant to show them how to use the app.

The app did not fit the nurses’ workflow, which involves toggling among multiple screens in the EHR. To access the vendor website, the PCP and nurses had to go outside of the EHR. Echoing the patients’ comments, the nurses and PCP said that the app was “one more thing to manage”. The nurses reported “a lot of uneasiness” about messaging with patients, who, despite being told that staff would respond within 24 hours to a post, wanted an immediate response the nurses could not provide. The nurses and PCP also noted that patients often do not follow through with a plan of care and so were not surprised that patients didn’t use the app as directed.

Patients have their own challenges, health is not their first priority…maybe the app was asking them too much.

##### Interface Dimension

When asked to describe the ideal app, the comments of the nurses and provider echoed those of the patients. They want something that interfaces with the EHR, providing a single point of entry into patient information.

The technology needs to be easy to use, for programs to talk to each other and give us the information we need instead of us searching for it….[there is too much] flipping [between screens], I want a whole picture in an easier way, not searching.

## Discussion

### Principal Findings

We did not test patients’ eHealth literacy before beginning the trial and lack of proficiency regarding technology and chronic disease in particular may have reduced the usability of the app for patients who enrolled and dissuaded patients who were not interested. While we were fairly confident that most patients had a mobile phone, many had phones that did not support apps and those who did have mobiles phones with app capabilities were not proficient at using apps. Finally, motivation for behavior change amongst eligible patients may have been lower than we had hoped. Most had had poorly managed disease for some time and getting their chronic condition in better control did not rise to the level of urgency for them that it did for the PCP and nurses.

The recruitment approach did not fit the workflow of clinical staff as originally planned. There was not enough time in a 20-minute visit to explain the app to patients and assist with downloading it if needed. Patients left promptly following their visits and phone calls made by research assistants were not returned.

In other trials using this app [13,14], the treatment approach was to assign patients to a nurse or health coach whose job was to track and correspond with patients using the app. However, the PCP and 2 nurses at CHCI did not have dedicated time to devote solely to these activities. Rather, our treatment approach was to incorporate the app into routine practice.

In general there was a poor fit between the app, the end-users, the recruitment, and the treatment approaches in our setting. Additional usability testing might have revealed this fact prior to launch. For both patients and staff, the app became an add-on, just one more thing to attend to. Lack of integration with the EHR required staff to toggle between internal and external platforms, which took time and multiple steps. It also might have helped if staff had dedicated time to work specifically with the patients using the app; but as an unreimbursed activity, such work was not feasible. Consequently, the key feature of the app—collaboration between the healthcare team and patient—could not be achieved.

### Conclusion

This brief trial underscores some of the pitfalls faced by providers hoping to utilize mHealth apps to improve chronic disease outcomes for some medically underserved patients. Effective use of mHealth tools for clinical management requires a good fit between the app, the users’ eHealth literacy, recruitment efforts, the treatment approach, and resources, especially time and reimbursement for services [5-8,20]. These last factors—treatment approach, time, and reimbursement—are contextual factors of the healthcare setting that affected the adoption of the app in this brief trial, and context is a significant factor in implementation efforts [21]. While the eUTC is placed within the context of healthcare, we recommend that future researchers be more explicit about contextual factors in the trial and adoption of mHealth technologies in addition to factors related to the end-users.

## Abbreviations

CHCI: Community Health Center, Inc.
eHealth: electronic health
EHR: electronic health record
eUTC: user-task-context
FBG: fasting blood glucose
FQHC: Federally Qualified Health Center
HbA1c: glycated hemoglobin
MA: medical assistant
mHealth: mobile health
PCP: primary care provider
RN: registered nurse
UDS: Uniform Data System
